# Clinical and MRI features associated with remission following biological therapy in axial spondyloarthritis

**DOI:** 10.1007/s10067-026-08380-9

**Published:** 2026-09-11

**Authors:** Ana Belén Azuaga, Ana Isabel García-Díez, Claudia Arango, Jaime Isern-Kebschull, Beatriz Frade-Sosa, Juan C Sarmiento-Monroy, Marina Urbano, Juan D Cañete, Julio Ramírez

**Affiliations:** 1 Arthritis Unit, Rheumatology Department, Hospital Clínic, Villarroel, Barcelona, 170, 08036 Spain; 2 Radiology Department, Musculoskeletal Unit, Hospital Clínic, Barcelona, Spain; 3 University of Barcelona, Barcelona, Spain; 4 Institut d’Investigacions Biomèdiques August Pi I Sunyer (IDIBAPS), Barcelona, Spain

**Keywords:** Axial spondyloarthritis, ASDAS, Biologic therapy, MRI, Remission

## Abstract

**Objective:**

To evaluate clinical and MRI features associated with clinical response in patients with axial spondyloarthritis (axSpA) initiating biological therapy.

**Methods:**

Forty-one biologic-naïve axSpA patients underwent baseline sacroiliac joint MRI before starting biological therapy. Clinical, inflammatory markers, disease activity scores, and MRI data (SPARCC score) were collected. Outcomes included clinical responses according to BASDAI and ASDAS.

**Results:**

Twenty-two were women (53.7%), with a median age of 40 years (IQR 34–55) and a median disease duration of 96 months (IQR 60–144). A total of 74.4% were HLA-B27 positive and all of them were clinically active, with median BASDAI and ASDAS-CRP scores prior to MRI of 6.4 (IQR 5.30–7.15) and 3.4 (IQR 2.98–4.03), respectively. At 16 weeks, 52.4% achieved BASDAI remission, which was independently associated with higher iliac erosion (OR 1.1, p 0.029). Clinically important ASDAS improvement occurred in 71.4% of patients and was significantly associated with higher baseline CRP (p 0.011). Major ASDAS improvement was linked to higher initial disease activity and iliac erosion scores, though not independently after adjustment. A combined baseline MRI score (bone edema + erosion) was significantly associated with achieving ASDAS inactive disease (AUC 0.74); No MRI or clinical variables retained independent predictive value in adjusted models.

**Conclusion:**

Higher iliac erosion scores are independently associated with BASDAI remission after biologic therapy in axSpA. A combined MRI inflammation and structural damage score may help predict ASDAS inactive disease. These findings highlight the value of MRI for identifying patients more likely to benefit from biologic therapy intervention.

**Table:** 

**Key Points** ∙ *Baseline MRI-detected iliac erosions identify patients more likely to achieve BASDAI remission after initiation of biologic therapy in axSpA.* ∙ *Patients achieving rapid and deep clinical responses show higher baseline MRI scores reflecting both active inflammation (BME) and established structural damage (iliac erosions).* ∙ *A combined MRI score integrating bone marrow edema and erosions improves prediction of ASDAS inactive disease, supporting the value of assessment of both inflammation and structural damage.*

## Introduction

Axial spondyloarthritis (axSpA) is a chronic inflammatory disease that primarily affects the spine, sacroiliac joints and entheses [1]. It is part of the spondyloarthritis spectrum, which also includes conditions such as psoriatic arthritis or reactive arthritis. AxSpA is classified into radiographic (r-axSpA) and non-radiographic (nr-axSpA) forms. The non-radiographic form represents earlier disease stages, in which structural changes are not yet detectable on conventional radiographs but can already be identified by magnetic resonance imaging (MRI), although part of these patients never progresses to ankylosing spondylitis (AS) [2, 3].

Unlike peripheral arthritis, the evaluation of axial involvement is more complex, and imaging techniques, particularly MRI, are important for diagnosis. Although bone marrow edema (BME) on MRI is an inflammatory finding commonly associated with axSpA and represents the key imaging criterion in the Assessment in SpondyloArthritis International Society (ASAS) classification criteria [4–6], it may also occur in other conditions, such as mechanical or disc disease, potentially leading to misdiagnosis [7]. Consequently, the presence of BME alone may be insufficient to establish either the diagnosis or the indication for treatment. The anatomical distribution of BME can help differentiate axSpA from mechanical conditions, as mechanical overload is typically associated with BME in the sacral portion of the sacroiliac joint and is usually more limited in extension compared with axSpA [8, 9].

After the acute inflammatory phase, reparative processes can also be detected on MRI. In this setting, BME is replaced by fat metaplasia (FM), and erosions may be refilled with fatty tissue, a phenomenon known as backfill (BF), which reflects post-inflammatory repair [10, 11]. Structural changes including erosions and, in advanced stages, sclerosis and ankylosis, further enhance the specificity of SpA diagnosis [12–14].

In patients with purely axial involvement, first-line management consists of physical exercise and nonsteroidal anti-inflammatory drugs (NSAIDs). In refractory cases, biological therapies or targeted synthetic disease-modifying antirheumatic drugs (DMARDs) are recommended [15–18]. In routine clinical practice, the most widely used biological DMARDs are tumor necrosis factor inhibitors (TNFi) and interleukin-17 inhibitors (IL-17i), whereas JAK inhibitors are also recommended in axSpA patients < 65 years without cardiovascular risk factors. All these advanced therapies are recommended for both r-axSpA and nr-axSpA [18]. Monitoring and assessing treatment response is strongly recommended using the Ankylosing Spondylitis Disease Activity Score (ASDAS), and when this is not feasible, the Bath Ankylosing Spondylitis Disease Activity Index (BASDAI) may be used [18]. The goal of treatment is to achieve remission or an improvement in ASDAS (ASDAS ≥ 1.1) after 12 weeks of treatment [18].

Despite advanced therapies, treatment response remains heterogeneous and has been linked to the presence of BME in the sacroiliac joints detected by MRI [5]. While BME is considered a predictor of therapeutic response [19, 20], its quantification and specificity within the context of axSpA remain uncertain. For this reason, the present study aims to evaluate clinical and imaging MRI findings for the prediction of remission/low disease activity 16 weeks after the onset of advanced therapies.

## Methods

### Study design

Single-center, exploratory retrospective study. This study was conducted in accordance with the principles of the Declaration of Helsinki and was approved by the Ethics Committee of the Hospital Clinic of Barcelona (HCB/2024/0761).

### Patients

We included patients older than 18 years with a diagnosis of axSpA under follow-up at the rheumatology outpatient clinics of our hospital. Patients were required to meet the ASAS classification criteria for axSpA [5], have no prior exposure (naïve) to biologic therapy or targeted synthetic DMARDs, and to have a sacroiliac joint MRI within the 6 months prior to biological DMARD initiation.

Clinical and laboratory data were collected at the visit preceding the start of advanced therapy (coinciding with the MRI date) and at 16 weeks after initiation of biologic or targeted synthetic DMARDs. Clinical data included demographic characteristics (sex, age, weight, height, body mass index, disease duration of axSpA, smoking status, previous treatments, patient-reported pain and fatigue VAS), laboratory parameters (C-reactive protein (CRP) and erythrocyte sedimentation rate (ESR)), and disease activity indexes (BASDAI and ASDAS-CRP).

Low disease activity was defined as BASDAI < 4 or ASDAS-CRP < 2.1 after treatment. Remission was defined as BASDAI < 2 or ASDAS-CRP < 1.3. Clinical improvement was defined as a reduction of ≥ 2 points in BASDAI or ≥ 1.1 points in ASDAS. Major clinical improvement was defined as a reduction of ≥ 2 points in ASDAS within 16 weeks after starting biologic or targeted synthetic DMARD therapy [21–23].

### Sacroiliac joint MRI assessment

Each image was divided into four quadrants (upper right sacrum and ilium, lower right sacrum and ilium, upper left sacrum and ilium, and lower left sacrum and ilium = 8 areas per slice). For the two most anterior slices, if the joint height was < 3 cm, only the two upper quadrants (right sacrum and ilium and left sacrum and ilium = 4 areas) were considered. In posterior slices, quadrants containing ligamentous portions were excluded.

Structural lesions were assessed in 5 oblique coronal slices on T1-weighted images (points per slice): FM (> 1 cm) (0–8), erosion (0–8), BF (0–4), ankylosis (0–4), and sclerosis (> 5 mm) (0–8).

BME was assessed in 6 oblique coronal slices on STIR images (points per slice): presence in each quadrant/bone/side (0–8), intense signal in right and left sacroiliac joints (0–2), and extensive involvement (> 1 cm) in right and left sacroiliac joints (0–2) [24–26].

In addition, a combined score integrating BME + erosion was calculated, as these lesions are most consistently linked with disease activity and structural damage in axSpA.

The overall interobserver reliability (kappa coefficient) was excellent (*κ* = 0.810; 95% CI 0.76–0.87; *p* < 0.001). The kappa values for each MRI feature were good for BME (*κ* = 0.667; 95% CI 0.52–0.81; *p* < 0.001) and for erosion (*κ* = 0.717; 95% CI 0.58–0.86; *p* < 0.001); and excellent for BF (*κ* = 0.906; 95% CI 0.79–1.00; *p* < 0.001), FM (*κ* = 0.805; 95% CI 0.66–0.95; *p* < 0.001), sclerosis (*κ* = 0.851; 95% CI 0.72–0.98; *p* < 0.001), and ankylosis (*κ* = 1.00; 95% CI 1.00–1.00; *p* < 0.001). Kappa coefficients were interpreted as follows: < 0.00 = none, 0.00–0.20 = poor, 0.21–0.40 = fair, 0.41–0.60 = moderate, 0.61–0.80 = good, and 0.81–1.00 = excellent.

### Statistical analysis

Patient demographics and disease characteristics were analyzed using standard descriptive statistics. Categorical data are presented as percentages, while continuous variables were reported as median and interquartile range (IQR). To compare quantitative variables, we used non-parametric Wilcoxon test, and for correlations Spearman’s rho. For the multivariate analysis, logistic regression models were used to assess the independent association between MRI findings and categorical outcomes (BASDAI remission and ASDAS response categories), while linear regression models were applied for continuous outcomes. All multivariate models were adjusted for age and disease duration. Results are reported as odds ratios (OR) with 95% confidence intervals (CI).

To assess the diagnostic utility of MRI in the identification of patients with clinical response, sensitivity, specificity, and positive and negative predictive values were calculated. Additionally, a receiver operating characteristic (ROC) curve and the area under the curve (AUC) were determined. The statistical analysis was performed using SPSS Statistics 27 (Chicago, IL, USA).

## Results

### Clinical and demographic features

A total of 41 patients were included in the study. Twenty-two were women (53.7%), with a median age of 40 years (IQR 34–55) and a median disease duration of 96 months (IQR 60–144). Seventeen patients (41.5%) were current smokers, and 19 (47.5%) were overweight or obese. Comorbidities included psoriasis in 5 patients (12.2%), uveitis in 10 (24.4%), inflammatory bowel disease (IBD) in 3 (7.3%), and fibromyalgia in 6 (14.6%). Enthesitis and peripheral arthritis were present in 11 (26.8%) and 13 (32.5%) patients, respectively. HLA-B27 positivity was found in 29 patients (74.4%). All patients were biologic-naïve at the time of MRI. Eight patients (20.5%) had received csDMARDs. Fourteen patients (37.8%) had CRP levels > 0.4 mg/dL. All patients were clinically active, with median BASDAI and ASDAS-CRP scores prior to MRI of 6.4 (IQR 5.3–7.1) and 3.4 (IQR 2.9–4.0), respectively. Table 1 summarizes the clinical and demographic characteristics before treatment.

**Table 1:** Clinical and demographics characteristics of the cohort

Age, median (IQR)	40 (34–55)
Female, *n* (%)	22 (53.7)
Current Smoker, *n* (%)	17 (41.5)
BMI, median (IQR)	24.20 (22.74–26.52)
Obese/overweight, *n* (%)	19 (47.5)
Month of evolution of SpA, median (IQR)	96 (60–144)
Radiographic SpA, *n* (%)	34 (82.9)
HLAB27 positive, *n* (%)	29 (74.4)
Peripheral arthritis, *n* (%)	13 (32.5)
IBD, *n* (%)	3 (7.3)
Uveitis, *n* (%)	10 (24.4)
Fibromyalgia, *n* (%)	6 (14.6)
ESR pretreatment (mmh), median (IQR)	9.00 (5.50–18.00)
CRP pretreatment (mg/dL), median (IQR)	0.40 (0.30–0.80)
BASDAI pretreatment, median (IQR)	6.40 (5.30–7.15)
ASDAS-CRP pretreatment, median (IQR)	3.45 (2.98–4.03)

*BMI* body mass index, *IQR* interquartile range, *HLA-B27* human leukocyte antigen B27, *IBD* inflammatory bowel disease, *ESR* erythrocyte sedimentation rate, *CRP* C-reactive protein, *BASDAI* Bath Ankylosing Spondylitis Disease Activity Index, *ASDAS-CRP* Ankylosing Spondylitis Disease Activity Score–C-reactive protein

### MRI findings at baseline

Median BME and erosion scores were 22 (IQR 5–27.5) and 15 (IQR 6–21.5), respectively. BME score was slightly higher in the iliac bone (median 6, IQR 1.2–10.5) than in the sacrum (median 5, IQR 2–9). Three patients showed no BME on MRI.

**Fig. 1:**
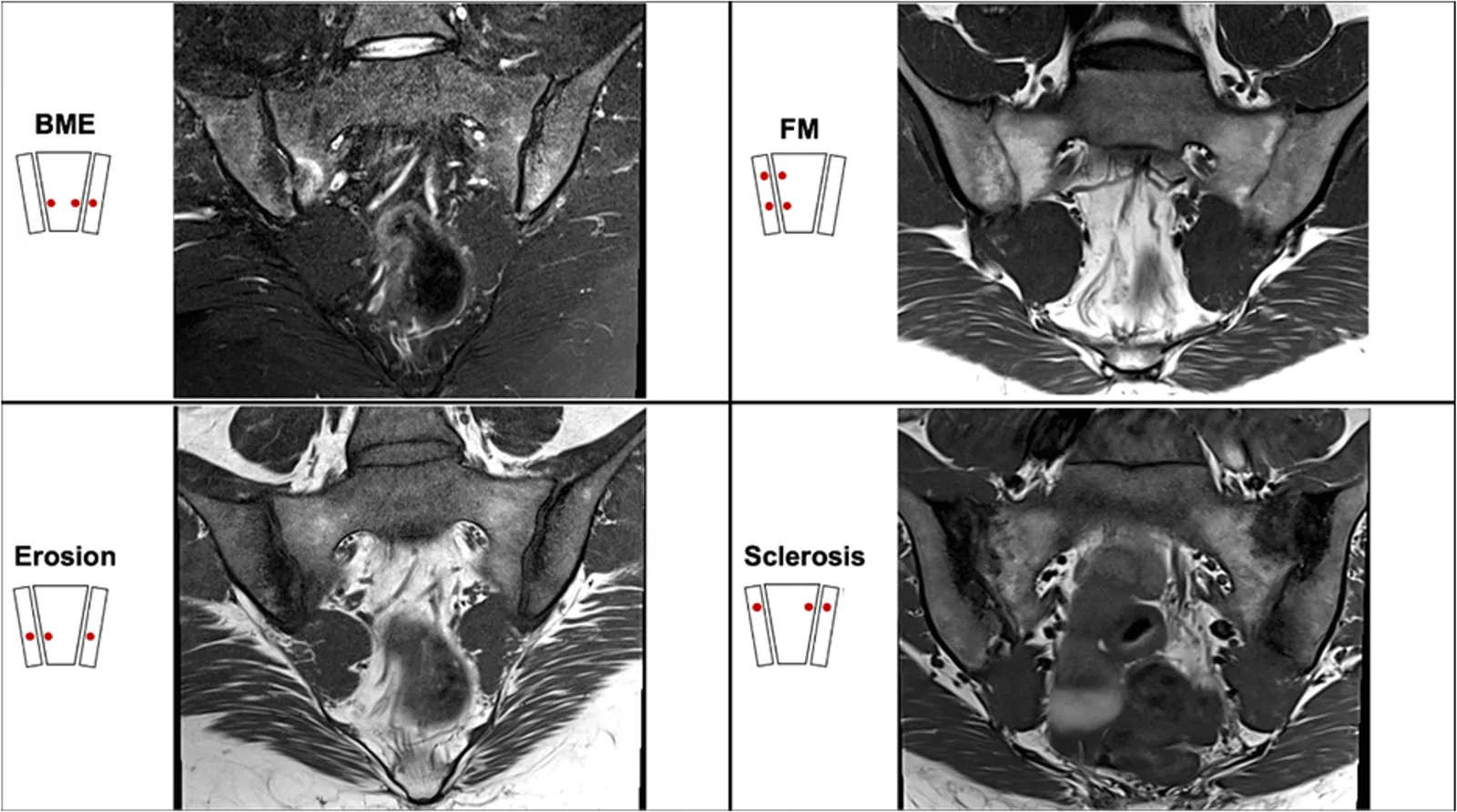
MRI findings and their locations. BME: Oblique coronal STIR image showing bone marrow edema in the inferior right sacrum and in the inferior left ilium and sacrum (3 points), with more intense involvement in the right sacrum (1 additional point). Erosion: Oblique T1-weighted image showing erosions in the inferior right ilium and sacrum, as well as in the inferior left ilium (3 points). Erosions were more pronounced in the right ilium. FM: Oblique T1- weighted image showing fat metaplasia (> 1 cm) in the superior and inferior regions of the right ilium and in the sacrum (4 points). Fat metaplasia was more prominent in the right sacrum. Sclerosis: Oblique T1- weighted image showing sclerosis (> 5 mm) in both the superior ilium and the left superior sacrum (3 points). BME: Bone Marrow Edema; FM: Fat Metaplasia

Structural lesions were commonly observed. Individual scores for BME and structural lesions are shown in Table 2. All but two patients had at least one erosion on MRI, predominantly in the iliac bone (median 12, IQR 6–15.7) compared to the sacrum (median 2, IQR 0–6.7). Other structural lesions were less frequent. Sclerosis was more prominent in the iliac bone, whereas FM was more commonly observed in the sacrum. Figure 1 shows the most common MRI findings and their locations.

**Table 2:** MRI scores

Scores	Total	Range
Bone marrow edema, median (IQR)	22.00 (5.00–27.50)	0–72
Erosion, median (IQR)	15.00 (6.00–21.50)	0–40
Fat metaplasia, median (IQR)	0 (0.00–3.50)	0–40
Sclerosis, median (IQR)	0 (0.00–6.00)	0–40
Backfill, median (IQR)	0 (0.00–2.00)	0–20
Ankylosis, median (IQR)	0(0–0)	0–20
Total MRI, median (IQR)	44.00 (19.00–57.00)	0–232
Total structure, median (IQR)	19.50 (10.00–33.250)	0–160

*IQR *interquartile range, *MRI *magnetic resonance imaging

No correlations were found between MRI findings (BME and erosions) and disease activity indexes (BASDAI and ASDAS-CRP) at baseline.

### Longitudinal follow-up

Following the baseline sacroiliac joint MRI, all patients initiated biological therapy according to local guidelines and clinical judgment. Of these, 34 patients (82.9%) received TNFi, and 7 (17.1%) received IL-17i.

**Fig. 2:**
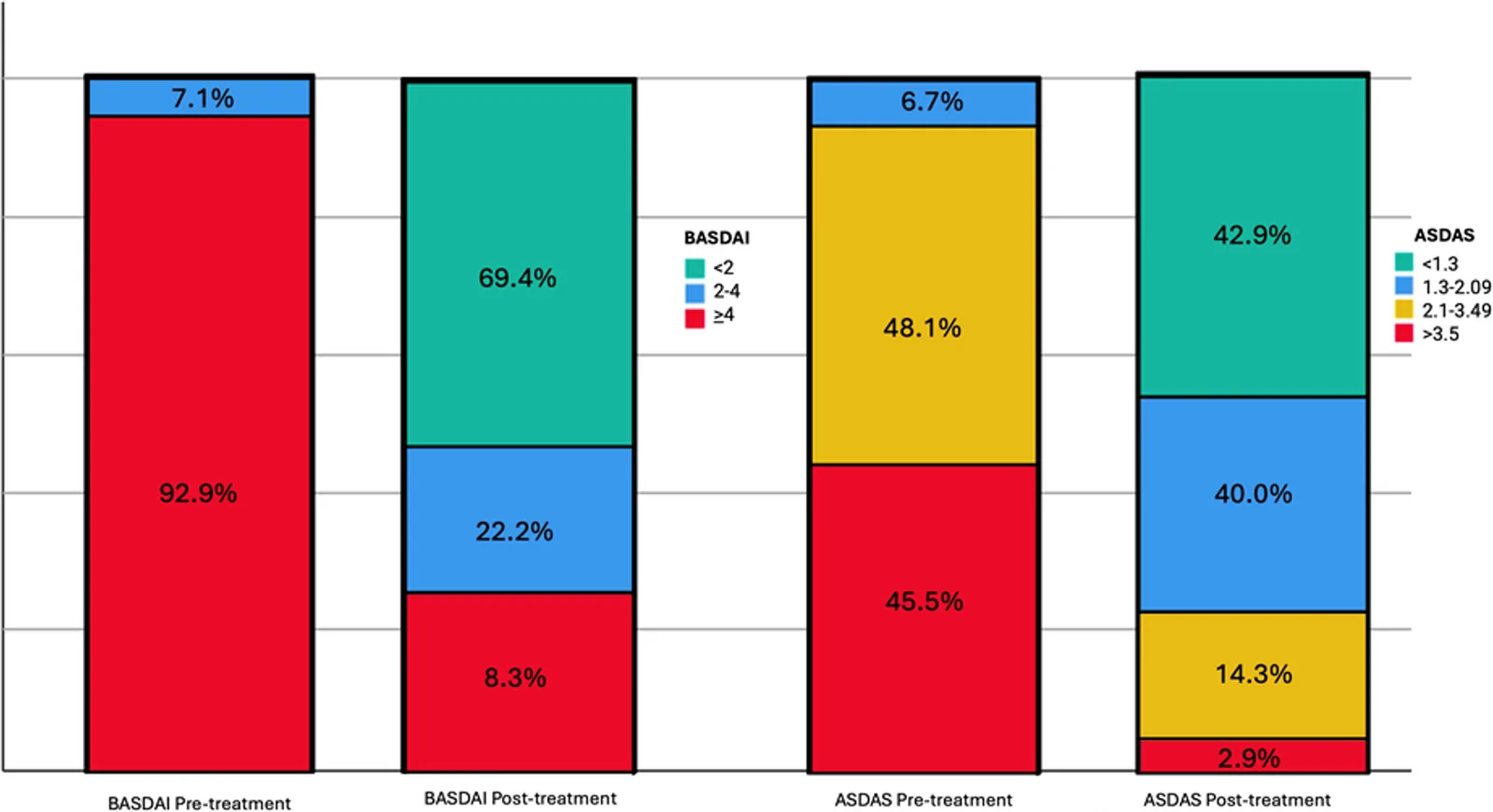
Distribution of disease activity states according to BASDAI and ASDAS before and after treatment. After treatment, a clear shift toward lower disease activity categories is observed for both indices, reflecting an overall improvement in clinical disease activity. BASDAI, Bath Ankylosing Spondylitis Disease Activity Index; ASDAS, Ankylosing Spondylitis Disease Activity Score

After 16 weeks, all disease activity scores (BASDAI and ASDAS) showed significant improvement. Figure 2 illustrates the changes in clinical indices before and after treatment. Twenty-two patients (52.4%) achieved BASDAI remission (< 2). Thirty patients (71.4%) experienced a clinically important improvement (≥ 1.1), and 14 (33.3%) achieved a major improvement (≥ 2) according to ASDAS criteria. Additionally, 29 patients (69%) reached low disease activity (ASDAS < 2.1), and 19 (45.2%) achieved inactive disease (ASDAS < 1.3).

**Fig. 3:**
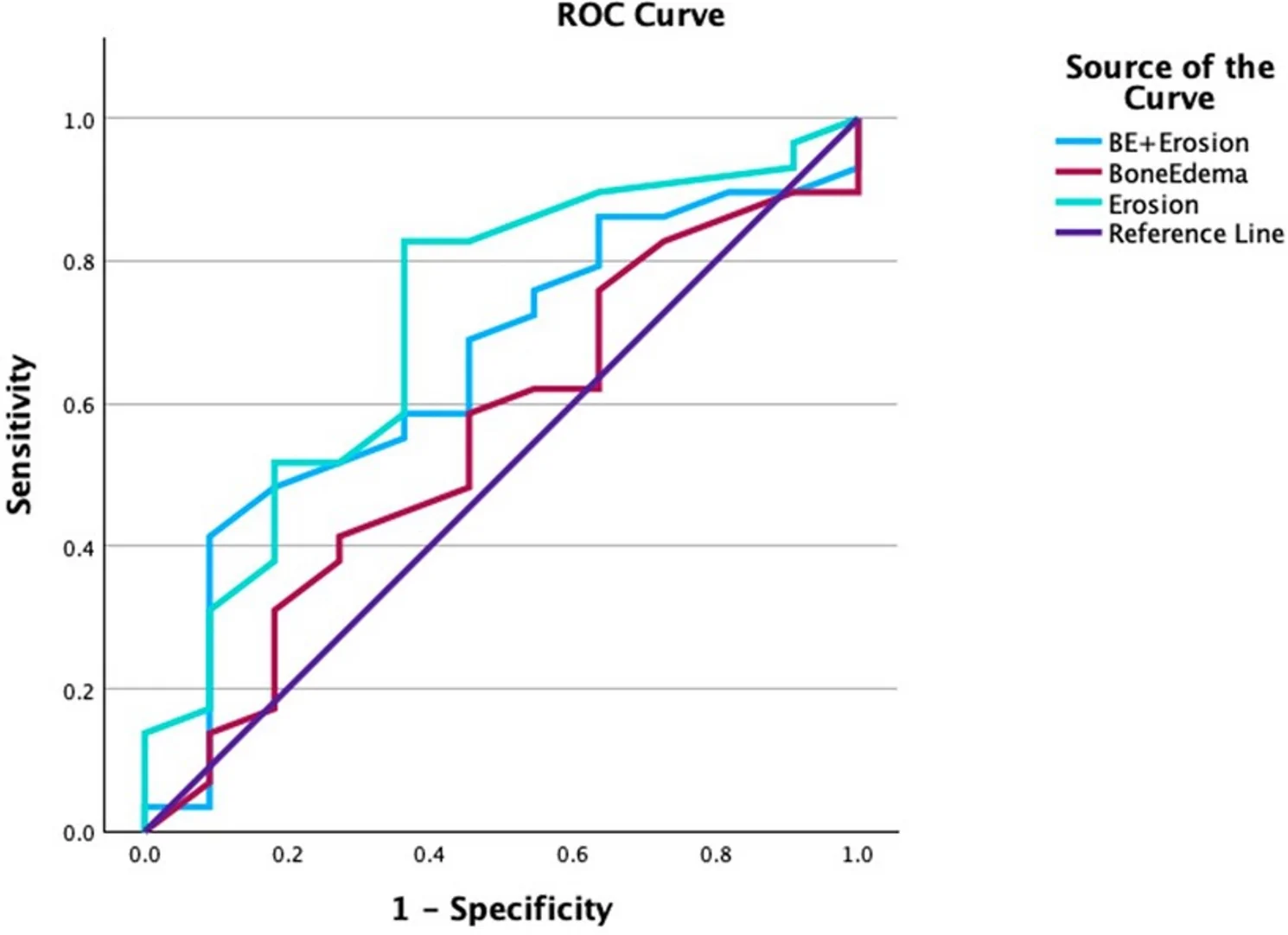
ROC curves evaluating the diagnostic performance of bone marrow edema, erosion, and their combination (bone marrow edema + erosion) for BASDAI remission. Sensitivity is plotted against 1 − specificity. The diagonal line represents the reference line corresponding to no discriminative ability (AUC = 0.5). ROC: Receiver operating characteristic; BASDAI, Bath Ankylosing Spondylitis Disease Activity Index; AUC, area under the curve

The 22 patients (52.4%) who achieved BASDAI remission had significantly higher erosion scores on MRI (*p* = 0.015), specifically in the iliac bone (*p* = 0.009), but not in the sacrum. Nearly all patients with psoriasis (4/5), uveitis (9/10), and IBD (3/3) reached BASDAI remission. In contrast, only one out of six patients with fibromyalgia reached this goal. Most patients with peripheral involvement (enthesitis and arthritis) were in BASDAI remission after 3 months, though the small number limited statistical significance. In multivariate analysis adjusted for age and disease duration, erosion score remained independently associated with BASDAI remission (OR 1.1, 95% CI 1.0–1.2, *p* = 0.029). A ROC curve analysis showed that MRI erosion score could identify up to 73% of patients achieving BASDAI remission (Fig. 3). Notably, all patients with an erosion score ≥ 24.5 achieved remission after starting biologic therapy.

Thirty patients (71.4%) achieved a clinically important improvement in ASDAS (≥ 1.1). They had significantly higher baseline CRP levels (*p* = 0.011). Similarly, all patients with psoriasis, uveitis, or IBD, and nearly all with peripheral involvement reached this goal. However, no baseline MRI findings were associated with this outcome. In multivariate analysis adjusted for age and disease duration, neither clinical nor MRI findings remained independently associated with ASDAS clinically important improvement: CRP levels (OR 85.6, 95% CI 0.4–15,201.6, *p* = 0.09).

Fourteen patients (33.3%) reached major ASDAS improvement (≥ 2). They had significantly higher baseline disease activity (BASDAI, *p* = 0.002; ASDAS, *p* = 0.002). Interestingly, these patients also had significantly higher iliac erosion scores at baseline MRI (*p* = 0.026). In multivariate analysis adjusted for age and disease duration, neither clinical nor MRI findings remained independently associated with this outcome: BASDAI, (OR 2.1, 95% CI 0.7–5.9, *p* = 0.1); ASDAS, (OR 9.1, 95% CI 0.8–96.5, *p* = 0.06); Iliac erosion score, (OR 1.0, 95% CI 0.8–1.3, *p* = 0.5).

Twenty-nine patients (69%) who reached low disease activity (ASDAS < 2.1) had significantly higher baseline ASDAS score (*p* = 0.039). No other clinical factors were associated with this outcome. Regarding MRI findings, trends were observed for BME (*p* = 0.086) and erosion score (*p* = 0.067), but only their combined score (BME + erosion) reached statistical significance (*p* = 0.043). All these factors lost their significance after adjusted analysis by age and time of evolution: ASDAS, (OR 0.21, 95% CI 0.0–1.5, *p* = 0.1); BME + erosion score, (OR 1.0, 95% CI 0.9–1.1, *p* = 0.1).

**Fig. 4:**
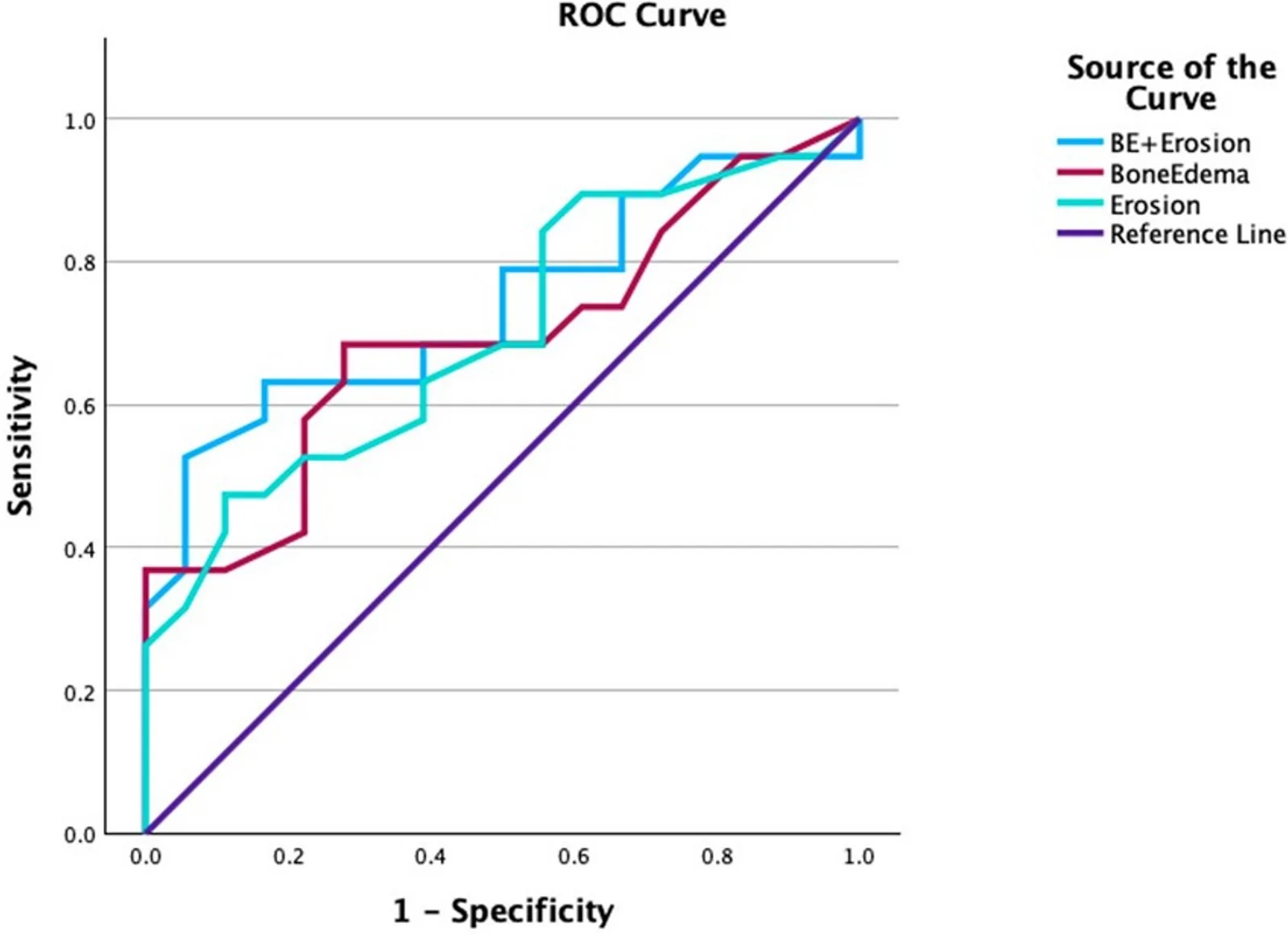
ROC curves showing the diagnostic performance of bone marrow edema, erosion, and their combination (bone marrow edema + erosion) for ASDAS inactive disease. Sensitivity is plotted against 1 − specificity. The diagonal line represents the reference line indicating no discriminative ability (AUC = 0.5). ROC, receiver operating characteristic; ASDAS, Ankylosing Spondylitis Disease Activity Score; AUC, area under the curve

Finally, 19 patients (45.2%) achieved ASDAS inactive disease (< 1.3) after 16 weeks of biological therapy. These patients had a longer disease duration (*p* = 0.036). Both BME (*p* = 0.046) and erosion scores (*p* = 0.036) at baseline MRI were significantly higher in this group. However, in multivariate analysis adjusted for age and disease duration, no factors remained independently associated with ASDAS inactive disease: disease duration, (OR 1.0, 95% CI 0.9–1.0, *p* = 0.83); BME, (OR 1.0, 95% CI 0.9–1.1, *p* = 0.19); erosion score, (OR 1.0, 95% CI 0.9–1.1, *p* = 0.43). A ROC curve model including baseline erosion and bone edema scores showed that up to 74% of patients achieving ASDAS inactive disease could be identified (Fig. 4). Remarkably, 94.4% of patients with a combined BME + erosion score ≥ 43 reached inactive disease. All patients with a BME score ≥ 27.5 or erosion score ≥ 22.5 achieved ASDAS inactive disease after starting biological therapy.

## Discussion

In this study, we evaluated the clinical and imaging characteristics of patients with axSpA prior to the initiation of biological therapy and assessed the association of baseline MRI findings with short-term clinical outcomes. Our results demonstrate that BME and structural damage, particularly erosions quantified by MRI, may serve as a predictor of clinical response, especially in relation to BASDAI remission and ASDAS inactive disease.

A key finding of our analysis is the strong association between iliac bone erosion scores and the achievement of BASDAI remission during follow-up after treatment. Patients with higher erosion scores, particularly in the iliac region, were significantly more likely to reach clinical remission, and all patients with erosion scores > 24.5 achieved this outcome. These results suggest that structural damage detectable by MRI may identify a subset of patients with a more active or established inflammatory phenotype who are more likely to respond favorably to biological therapy. This finding aligns with previous observations that structural lesions at MRI, such as erosions and FM, reflect cumulative inflammatory activity and may coexist with active inflammation [4, 19, 27, 28]. However, it is important to acknowledge that this association may not reflect a direct predictive role of structural damage per se, but rather a confounding effect of underlying disease severity: patients with more advanced inflammatory disease tend to accumulate greater structural lesions over time and, simultaneously, may exhibit a more pronounced clinical response once effective biological therapy is initiated. In this context, erosions would serve as a surrogate marker of cumulative inflammatory burden rather than an independent driver of therapeutic response, a distinction that prospective studies with larger cohorts would be needed to confirm.

The ASAS classification criteria for axSpA include only inflammatory lesions [5]; however, structural lesions are also considered to contribute to the diagnosis, as they have high specificity for axSpA [4, 29]. More recently, a subanalysis of patients treated with TNFi therapy showed that those who met the MRI criteria based solely on inflammatory lesions (BME) in two slices had lower response rates to clinical outcomes compared with patients who also presented structural lesions and involvement in more slices [30].

Interestingly, we also observed that patients with comorbid psoriasis, uveitis, or IBD had high rates of clinical response, consistent with the notion that extra-musculoskeletal manifestations may indicate a more systemic inflammatory burden. In contrast, the presence of fibromyalgia was negatively associated with remission, reinforcing the challenge of differentiating inflammatory symptoms from overlapping pain syndromes in axSpA, which can attenuate the perceived efficacy of biological treatment [31, 32].

Furthermore, BASDAI is a patient-reported outcome measure that may be influenced by non-inflammatory factors such as fibromyalgia or altered pain perception, which could explain why MRI erosions independently predicted BASDAI remission but not ASDAS-based outcomes. Unlike BASDAI, ASDAS incorporates objective inflammatory markers such as CRP, making it less susceptible to subjective confounders [33, 34]. The lack of independent MRI predictors for ASDAS outcomes after adjustment may therefore reflect the multifactorial nature of objective treatment response, in which systemic inflammation plays a predominant role over structural imaging findings in this small cohort.

Our data suggest that a combined MRI score incorporating both BME and erosions may be associated with achieving ASDAS low disease activity and inactive disease. This integrated approach may better capture the interplay between active inflammation and cumulative structural damage, supporting recent efforts to develop composite imaging indices that correlate with treatment response [35, 36]. In this exploratory analysis, a combined score > 43 was associated with ASDAS inactive disease, and notably, all patients with BME > 27.5 or erosion scores > 22.5 reached this outcome. These cutoff values, however, require prospective validation in larger cohorts before clinical application. Therefore, quantifying both inflammation and structural changes at baseline may enhance prognostic stratification prior to initiating biological treatment. These findings suggest that treatment is more effective in patients with confirmed signs of axSpA, rather than in those with isolated BME, which may also occur in other pathological or physiological conditions.

From a therapeutic standpoint, the majority of patients (83%) received TNFi, while a smaller proportion received IL-17i. Although subgroup analysis was not powered to detect treatment-specific differences, no significant differences should be expected, since a recent clinical trial has shown similar results for TNFi and IL17i in both clinical and imaging outcomes [37]. Moreover, the high overall rate of clinical response suggests that early identification of patients with high MRI scores may help guide prompt initiation of effective biologic therapy.

This study has several strengths, including the use of quantitative MRI scoring systems, a well-characterized real-world cohort, and standardized disease activity assessments. However, several limitations should be acknowledged. The sample size was relatively small, and the follow-up period was short (16 weeks), which, while appropriate for early response assessment, may not capture the full prognostic value of structural MRI lesions such as erosions, which could have greater relevance for longer-term outcomes. In several adjusted models, the limited number of events relative to covariates led to unstable estimates, reflected in markedly wide confidence intervals. These results should be interpreted with caution. In addition, the use of BASDAI may have been influenced by non-inflammatory factors such as fibromyalgia. Furthermore, although no significant differences were expected between treatment groups, the heterogeneity in biological therapy (83% of patients receiving TNFi and 17% IL-17i) may have influenced response patterns and cannot be entirely excluded as a confounding factor. Future studies with larger cohorts and longer follow-up are warranted to validate these findings and to further assess the prognostic utility of MRI-derived scores in guiding individualized treatment strategies.

In conclusion, our findings suggest that MRI-detected iliac erosions and combined BME with erosion scores may serve as exploratory indicators of short-term clinical remission in axSpA, though validation in larger prospective cohorts is needed. Incorporating structural lesion assessment into baseline imaging evaluation may improve patient stratification and inform early therapeutic decisions in clinical practice.

## Data Availability

Full data is available upon reasonable request.
